# Direct Exposure to Outdoor Air Pollution Worsens the Functional Status of Stroke Patients Treated with Mechanical Thrombectomy

**DOI:** 10.3390/jcm13030746

**Published:** 2024-01-27

**Authors:** Anetta Lasek-Bal, Wiktor Rybicki, Sebastian Student, Przemysław Puz, Aleksandra Krzan, Aleksandra Derra

**Affiliations:** 1 Department of Neurology, School of Health Sciences, Medical University of Silesia in Katowice, 40-055 Katowice, Poland; ppuz@o2.pl (P.P.); aleks.krzan@gmail.com (A.K.); 2 Department of Neurology, Upper-Silesian Medical Centre of the Silesian Medical University in Katowice, 40-635 Katowice, Poland; wrybicki@gcm.pl (W.R.); oladerra@gmail.com (A.D.); 3 Faculty of Automatic Control Electronics and Computer Science, Silesian University of Technology, 44-100 Gliwice, Poland; sebastian.student@polsl.pl; 4 Biotechnology Center, Silesian University of Technology, 44-100 Gliwice, Poland

**Keywords:** stroke, thrombectomy, air pollution

## Abstract

Background The effect of air pollutants on the functional status of stroke patients in short-term follow-up is unknown. The aim of this study was to evaluate the effect of air pollution occurring in the stroke period and during hospitalization on the functional status of patients undergoing mechanical thrombectomy (MT). Methods Our study included stroke patients for which the individual-level exposure to ambient levels of O_3_, CO, SO_2_, NO_2_, PM2.5, and PM10 during the acute stroke period was assessed. The correlations between the air pollutants’ concentration and the patients’ functional state were analyzed. A total of 499 stroke patients (mean age: 70) were qualified. Results The CO concentration at day of stroke onset was found to be significant regarding the functional state of patients on the 10th day (OR 0.014 95% CI 0–0.908, *p* = 0.048). The parameters which increased the risk of death in the first 10 days were as follows: NIHSS (OR 1.27; 95% CI 1.15–1.42; *p* < 0.001), intracranial bleeding (OR 4.08; 95% CI 1.75–9.76; *p* = 0.001), and SO_2_ concentration on day 2 (OR 1.21; 95% CI 1.02–1.47; *p* = 0.03). The parameters which increased the mortality rate within 90 days include age (OR 1.07; 95% CI 1.02–1.13; *p* = 0.005) and NIHSS (OR 1.37; 95% CI 1.19–1.63; *p* < 0.001). Conclusions Exposure to air pollution with CO and SO_2_ during the acute stroke phase has adverse effects on the patients’ functional status. A combination of parameters, such as neurological state, hemorrhagic transformation, and SO_2_ exposure, is unfavorable in terms of the risk of death during a hospitalization due to stroke. The risk of a worsened functional status of patients in the first month of stroke rises along with the increase in particulate matter concentrations within the first days of stroke.

## 1. Introduction

Ischemic stroke (IS) is a multifactorial disease. The various modifiable factors that may contribute to stroke include smoking, lack of physical activity, substance abuse, air pollution, and transportation noise [1,2]. Acute exposure to air pollution increases the risk of vascular diseases, including stroke and death [3,4,5,6]. Mechanical thrombectomy (MT) has established its efficacy as a causative treatment for acute ischemic stroke with large vessel occlusion. To date, some independent factors regarding the prognosis of the functional status of patients after MT have been identified, including age, the neurological status on the first day of the stroke, as well as selected clinical parameters, including hyperglycemia on the first day of the stroke, chronic kidney disease, and atrial fibrillation [7,8,9,10,11]. Some authors have demonstrated the important role of air pollution on stroke morbidity and mortality, independent of the treatment methods of stroke [12,13,14].

Air pollutants are composed of particulate matter (a mixture of solid particles and liquids) and gaseous components. The results of previous studies indicate a close association between mortality and air dust content; however, a higher concentration of gases, such as carbon monoxide, nitrogen dioxide, and sulfur dioxide, have less influence on the prevalence of vascular diseases and mortality [15]. According to the published observations, the admission of patients to hospital for neurological diseases, including cerebral ischemia on dusty days, increased significantly compared to days when air pollution was low [16].

To date, a cause-and-effect relationship between air pollution and stroke incidence has been repeatedly proven. However, the effect of air pollutants on the acute phase of stroke or the post-stroke status of patients during short-term follow-up is unknown as of yet. The eye conjunctiva and the respiratory system as well as the gastrointestinal tract are the first barrier for air pollutants [17,18,19]. The particles responsible for air pollution were shown to have prothrombotic and proinflammatory effects [20]. Inflammatory mediators can enter the circulatory system, stimulate the release of coagulation factors, impair vascular function, and increase thrombosis, which can lead to mortality from vascular diseases [21]. In preclinical studies, the exposure to ambient dust particulate matter increased brain edema and blood–brain barrier permeability by increasing the inflammatory responses and oxidative stress [22].

Silesia is a very heavily industrialized region in southwestern Poland. Heavy industry is the highest contributor to air pollution when compared to other Polish cities. The incidence of stroke in the Silesia region is greater than that observed in other regions of Poland [23].

The aim of this study was to evaluate the effect of air pollution occurring over two days immediately preceding stroke symptoms and in the acute stage of stroke (the stroke onset day, the second, the fifth, and the eighth day), on the functional status on day 10 and day 90 in patients undergoing endovascular treatment at the Center for Interventional Stroke Treatment in Katowice, the Silesian Metropolis.

## 2. Methods

A retrospective study included patients treated with mechanical thrombectomy (MT) due to stroke in the Medical Centre at the Silesian Medical University in Katowice over the period of 42 months (2019–2022). All our patients, residents of Katowice or other Silesian cities who underwent MT in the period mentioned above, were initially enrolled in the study. The key inclusion criteria were as follows: first ever stroke, staying in Silesia during the acute stage of the disease, LVO stroke, and endovascular treatment. Those patients who were included in the study were chosen based on the analysis of the following criteria: age at the time of first ever stroke; present comorbidities, such as atrial fibrillation (AF), arterial hypertension (AH), coronary heart disease (CHD), diabetes mellitus (DM), lipid disorders (LD), and >70% atherosclerotic carotid artery stenosis according to the NASCET (North American Symptomatic Carotid Endarterectomy Trial) criteria (CAS, ipsilaterally to the acute ischemic brain lesion) [24]; neurological condition on the first and second day of stroke, as evaluated using the National Institute of Health Stroke Scale (NIHSS) [25]; functional status on days 10 and 90 following stroke as per the modified Rankin Scale (mRS) [26]; head computed tomography (CT) results at 24 h after MT.

We assessed individual-level exposure to ambient levels of ozone (O_3_), carbon monoxide (CO), sulfur dioxide (SO_2_), nitrogen dioxide (NO_2_), and particulate matter PM2.5 (PM2.5—particles with aerodynamic diameter <2.5 μm) and PM10 (PM10—particles with aerodynamic diameter <10 μm) in the study period.

The data on dust particle and gas air pollution in Katowice and adjacent cities were obtained from the Environmental Protection Inspectorate (state-owned institution), which measures, collects, and analyzes data and publishes them on its website [27]. The data collected related to air pollution in the areas where each patient stayed for two days directly before the stroke, on the day of stroke onset, and on days 5 and 8 of hospitalization.

AF was diagnosed on the basis of previous patient medical records/ECG or a 24 h ECG monitoring performed during hospitalization. Hemorrhagic lesions were evaluated based on head CT performed at 24 h after MT and the ECASS (European Cooperative Acute Stroke Study scale) [28].

Multivariable models were built using binary logistic regression for binary outcomes. The model variable selection procedures included automatic selection (stepwise, forward, and backward) based on the AIC (Akaike information criterion) and BIC (Bayesian information criterion) [29,30]. For the evaluation of the accuracy of model predictions, a “leave-one-out” procedure to avoid data leakage so as not to cause over-fitting and the AUC (area under the ROC curve) estimator were used. All statistical analyses were performed using R version 3.6.1.

The following parameters were analyzed using logistic regression: age; sex; AF; AH; CHD; DM; LD; >70% CAS; nicotinism; thrombolysis in cerebral infarct (TICI) scale; intracranial bleeding (ICB) after intervention, including symptomatic ICB, NIHSS, rtPA (recombinant tissue plasminogen activator); and the concentrations of O_3_, CO, SO_2_, NO_2_, PM2.5, and PM10 on the days prior to hospitalization (−2, −1), the day of stroke onset (0), and day 5 as well as day 8 of hospitalization.

We divided the pollution data (various contaminants) into four quartiles. In each quartile, we determined the frequency of poor functional outcomes (mRS > 2). We used the Pearson’s correlation coefficient test to check if the frequency of the poor functional outcomes grows or decreases monotonically. A *p*-value of less than 0.05 was considered statistically significant.

This study was approved by the local ethics committee (Bioethics Committee of the Medical University of Silesia in Katowice, PCN/CBM/0052/KB1/98/1/22 signed on 4 October 2022) in accordance with local and regional laws and was therefore performed in accordance with the ethical standards of the 1964 Declaration of Helsinki and its subsequent amendments. Due to the retrospective nature of the study, the informed consent of patients was waived in accordance with commission and local laws.

## 3. Results

**Figure 1:**
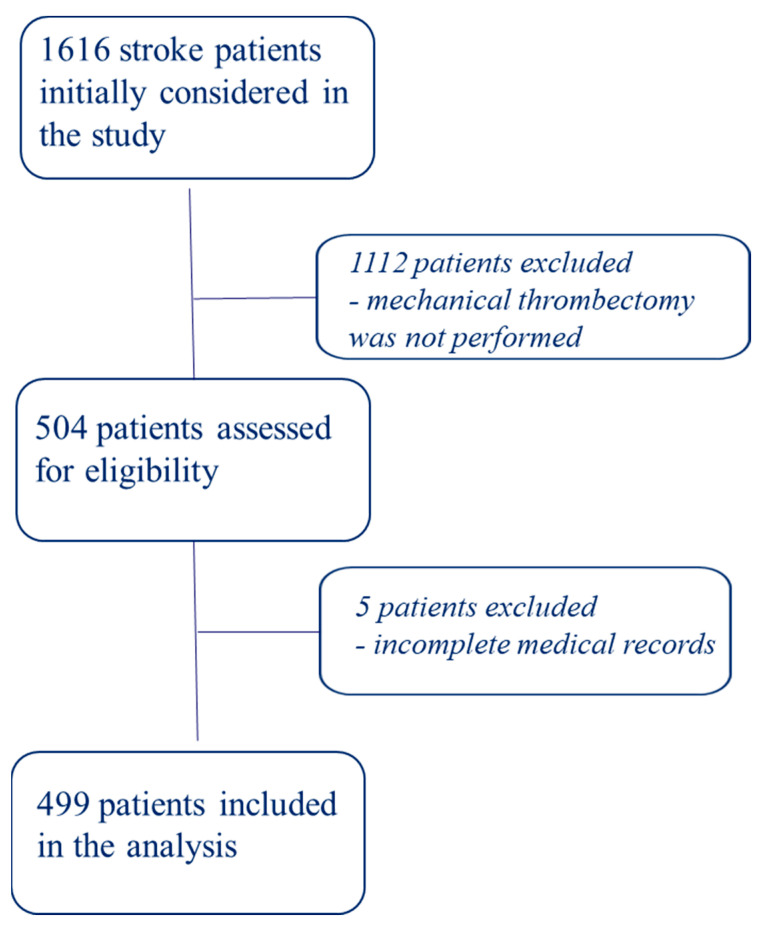
The flowchart of patient qualification.

A total of 504 stroke patients (mean age: 70 (19–92); female: 46.9%) hospitalized within 42 months (2019–2022) and treated with MT in the ultra-acute stroke period (≤6 h), were initially qualified for a retrospective study. The subjects made up 31.18% of all patients treated for ischemic stroke in the Upper-Silesian Medical Centre at the Silesian Medical University in Katowice in the above-mentioned period (1616 stroke patients). Additionally, we excluded five patients who underwent MT (full medical records were unattainable). Out of the 499 patients included, 317 (62.89%) were treated with recombinant tissue plasminogen activator (rtPA) + MT. The process of patient qualification is presented in Figure 1.

The characteristics of the patients enrolled in the study are shown in Table 1.

**Figure 2:**
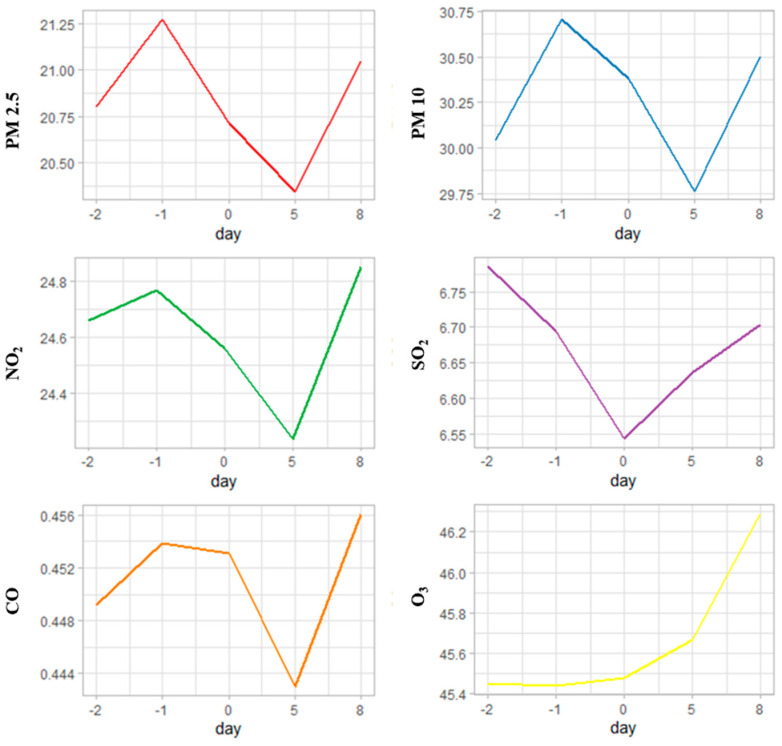
The mean concentration of gaseous components and particulate matter one day and two days before stroke, upon admission (stroke onset day), and on the 5th and 8th day of stroke. CO—carbon monoxide, NO_2_—nitrogen dioxide, O_3_—ozone, PM—particulate matter (PM2.5—particles with aerodynamic diameter <2.5 μm; PM10—particles with aerodynamic diameter <10 μm), SO_2_—sulfur dioxide.

The concentration of individual particles polluting the air was generally stable as was shown in the Figure 2. There was slight decrease in the SO_2_ concentration on the first day after onset as well as a decrease in concentrations of other gaseous components and particulate matter on the fifth day of stroke.

The fluctuations of air pollutant concentrations in the all study period are presented in the Supplementary Materials Tables S1 and S2.

Independent parameters of poor functional status according to the mRS on day 10 and day 90 after the onset were identified; these are shown in Table 2 and Table 3, respectively.

Among all study parameters influencing on the bad functional status on the 10th day of stroke, the independent effect had the parameters as follows: the neurological state on the 2nd day of stroke (according to NIHSS), TICI score, MT time, the burden of DM and/or LD and/or smoking as well as the air concentration of the CO.

The independent effect on the bad functional status on the 90th day of stroke had the parameters as follows: the age, the neurological state on the 2nd day of stroke (according to NIHSS), and the symptomatic cerebral bleeding in the CT of the head 24 h after MT (Table 4).

The receiver operating characteristic (ROC) curves of the regression model analysis of the impact phenodata on a worsened functional status of patients (>2 points on the modified Rankin Scale) on the 10th and 90th day of stroke are presented in the Supplementary Materials.

The independent parameters which increase the risk of death within 90 days of the onset include age (OR 1.07; 95% CI 1.02–1.13; *p* = 0.005) and NIHSS on day two (OR 1.37; 95% CI 1.19–1.63; *p* < 0.001).

The PM 2.5 pollution on the first day of stroke influenced on poor functional outcome of our patients at discharge. It was statistically significant with high positive correlation coefficient. Similarly, the incorrect concentrations of PM2.5 on the 5th day as well as the concentration of PM10 on the 8th day was important for bad functional outcomes on the 30th day. The results were statistically significant with high positive correlation coefficient (Table S3 in Supplementary Materials).

## 4. Discussion

The main result of the study is that regardless of a patient’s clinical profile, exposure during the acute period of stroke to air polluted with CO and SO_2_ had adverse effects on the course of stroke, and it worsened the prognosis in terms of a patient’s functional status and death within 10 days. However, no significant effect resulting from potential air pollution exposure during the acute stroke period was demonstrated on the functional status of patients and their prognoses for the subsequent three months.

In our study, both a severe neurological state and symptomatic intracranial hemorrhage increased the risk of death in the early days after the onset. Similarly, the neurological state of our patients and a patient’s older age had an unfavorable effect on the risk of death in the first three months after the onset of stroke.

Convincing evidence shows that exposure to air pollution is associated with an increasing risk of atherosclerosis, stroke, and heart failure [3,6,21]. The cardiovascular response to air pollution is modulated by the chemical composition of pollutants and their concentration at exposure, the duration of such exposure, individual vulnerability, comorbidities, and the changes in meteorological parameters, such as temperature, humidity and ambient pressure [31]. Exposure to air pollution has been associated with stroke hospitalization but the evidence of its effects on ischemic stroke is limited and inconsistent [15,16]. The type of air pollutants can be different according to geographical, social, and industrial circumstances. We decided to analyze the potential influence of the most common gases and particles on selected clinical aspects in stroke patients treated with MT. It is important to identify how air pollution or limited access to the natural environment contribute to stroke burden.

Some authors have investigated the association between chronic particulate matter exposure, incidental stroke, and stroke-related death. The pooled hazard ratio for each increase in PM2.5 levels by 5 μg/m^3^ was 1.11 (95% CI 1.05–1.17) for incidental stroke was the same for stroke-related death [32]. The presented study shows no relationship between PM pollution and the post-stroke functional status of patients. The inconsistencies may be due to some modifiable factors, like seasonal and meteorological parameters, as well as due to the patient’s clinical profile. Moreover, studies conducted on populations of various continents have brought varied results. A significant relationship between PM2.5 levels and incidental stroke has previously been found regarding Europe and North America, whereas a pooled result in Asia was insignificant [33]. The link between hypercoagulability and exposure to PM2.5 has been suggested, as well as the prothrombotic effect of soluble metal compounds (e.g., sulfate) [34,35,36,37,38].

According to the results obtained in this study, CO exposure proved to be significant for the functional status of patients; poor neurological condition, symptomatic intracranial hemorrhage, and exposure to SO_2_ pollution increased the risk of death in the acute period of stroke. The unfavorable effects of polluted air during the first hours of stroke can be demonstrated by several pathogeneses of risk factors. Such effects are most probably associated with an inflammatory process, oxidative stress, and autonomic dysfunction. Inhalation of SO_2_ can affect heart rate variability, increase oxidation, and exacerbate blood clotting and thrombosis formation [38,39,40,41].

In the presented study, an increase in the presence of CO in the air during the early days of stroke was an independent parameter influencing the functional status of acute stroke patients. In accordance with previous study results, exposure to air polluted with CO increased the risk of stroke. Our results indicate an additional adverse effect of gas during the course of the acute period of stroke. The concentration of CO in air is not deleterious for humans but, with continuous industrial developments, the use of coal and petroleum has increased CO emissions and has certainly impacted human health [42,43]. The latest results identified CO air pollution to be a new important risk factor for stroke [42,43,44]. This effect may be due to seasonal differences in CO exposure or CO air pollution [13]. Varied susceptibility associated with sex and age might be related to a patient’s lifestyle. The elderly with weaker immune systems and underlying chronic diseases might be more sensitive to air pollution exposure. A study conducted in Taiwan found that CO was significantly and positively associated with stroke hospitalizations in a single-pollutant model but insignificant in a multi-pollutant model [45]. Contrarily, a study conducted in Hong Kong did not find an association between ambient CO concentrations and stroke hospitalizations [46]. Inconsistent results pertaining to the association between CO and stroke might be attributable to variations, including air pollution levels, definitions of the outcomes, weather conditions, and susceptibility among certain populations. Regardless, the daily or seasonally changes in air pollutant concentrations can cause health damage [47].

Previous studies have proved that environmental NO_2_ exposure was associated with an increased risk of stroke in areas of heavy air pollution [48,49]. Ozone (O_3_) is a secondary pollutant with strong oxidizing properties [50]. In recent years, the concentrations of other pollutants, such as fine particulate matter (PM2.5), have decreased, whereas ambient O_3_ concentration is stable or has gradually increased globally [1]. In our study, exposure to NO_2_ or ozone was not found to be a factor that would independently influence the course of stroke. There is the presumed concentration-response curve threshold above which the effect of harmful pollutants is present.

According to our study, the combination of clinical factors and air pollution with SO_2_ and CO is significant for the functional status of stroke patients treated with MT.

The negative impact of the above-mentioned gases was observed in the acute period of stroke. Exposure to CO and SO_2_ may result in several pathophysiological changes related to the acute stage of stroke, such as local and systemic inflammation and a predisposition to cardiac arrhythmias. It is difficult to clearly state how exposure to these gases affects the functional state of patients in the chronic period of the stroke. According to the results of our study, the impact is neutral.

It is probable that the effect of air pollutants is modified by other variables (including comorbidities, emotional stress psychosocial parameters, temperature, traffic noise, and the synergistic effect of a mixture of pollutants). The potential effect may also be related to the concentration of the air pollutants during exposure. There is insufficient evidence concerning the health effects of exposure to pollutant concentrations lower than the current limits set by the European Union and World Health Organization. Independently, the data can vary by region of the world.

The World Health Organization has shown that approximately 20% of air pollution-related deaths are due to cerebral ischemia [51]. Another report revealed a downward trend in the mortality rate resulting from ischemic stroke attributed to exposure to air pollution; however, the data varied by world region [52]. According to the results of this study, mortality in the first three months was only influenced by age and the neurological condition in the first days of stroke.

Our results suggest an urgent need to control air pollution, especially in industrial regions and those with heavy road traffic. The identification of modifiable risk factors for stroke has substantial public health implications. As reported by Spanish researchers, residential developments surrounded by green areas were related to a lower incidence of IS [53]. It is also possible that greenery reduces the risk of a severe course of stroke; however, this area requires further research. Not all air pollutants are being monitored. There are countless potential pollutants, and new chemicals are constantly emerging as a result of interactions between existing ones. Therefore, most pollutants have never been assessed for toxicity and risk to stroke patients.

Our paper has some limitations. Firstly, it is retrospective. Next, it lacks any analysis of the meteorological parameters which can modify the influence of air pollution on a patient’s clinical status and the course of stroke. We did not consider the influence of other potentially relevant environmental determinants linked with the course of stroke, such as noise. In some cases, the information regarding air pollution exposition could be imprecise due to the potential migration of patients in the short time before stroke. The other limitation is the lack of individual air pollution measurements for each patient.

## 5. Conclusions

Exposure to air pollution with carbon monoxide and sulfur dioxide during the acute period of stroke has adverse effects on the acute phase of the disease and the post-stroke functional status of patients.

A combination of parameters, such as the neurological state of stroke patients, a hemorrhagic transformation of ischemic focus, and SO_2_ exposure, is unfavorable in terms of the risk of death during a hospital stay due to stroke.

Exposure to polluted air in the early days of stroke does not have a significant effect on the functional status of patients during the chronic period of disease.

The risk of a worsened functional status of patients in the first month of stroke rises, along with the increase in particulate matter concentrations within the first days of a stroke.

## Figures and Tables

**Table 1:** Characteristics of patients.

Parameter	Value
Age, mean, med., QR	67.53, 70, 16
Female, n (%)	239 (47.19)
NIHSS_1, med., IQR	13, 8
NIHSS_ 2, med., IQR	12, 8
mRS 8d, med., IQR	4, 2
mRS 90d, med., IQR	5, 4
rtPA iv (actylise), n (%)	309 (61.24)
MT time *, mean, SD	108.4, 42.24
TICI 2B-3, n (%)	339 (67.87)
ICB, n (%)	115 (22.7)
sICB, n (%)	25 (5)
Atrial fibrillation, n (%)	259 (51.67)
Arterial hypertension, n (%)	379 (76.12)
Diabetes mellitus, n (%)	122 (24.34)
Peripheral artery disease, n (%)	235 (47.14)
Nicotinism, n (%)	182 (36.48)
Lipid disorders, n (%)	209 (40.83)
Carotid artery stenosis, n (%)	53 (10.72)

* in minutes. ICB—intracranial bleeding, MT—mechanical thrombectomy, NIHSS—National Institutes of Health Stroke Scale; mRS—modified Rankin Scale, mRS 8d- mRS on the 8th day, mRS 90d—mRS on the 90th day of stroke, NIHSS_1—NIHSS on the 1st day of stroke before MT, NIHSS_2—NIHSS on the 2nd day of stroke, rtPA iv—intravenous recombinant tissue plasminogen activator, sICB—symptomatic ICB, TICI—thrombolysis in cerebral infarction.

**Table 2:** Binary regression analysis of the influence of clinical phenodata on a worsened functional status of patients (>2 points on modified Rankin Scale) on the 10th day of stroke.

Parameter	OR	95% CI	*p*
NIHSS_2	1.324	(1.204–1.472)	0.000
TICI	0.890	(0.811–0.967)	0.009
MT time	1.010	(1.001–1.021)	0.042
DM	4.341	(1.691–12.367)	0.004
Smoking	2.212	(1.064–4.712)	0.035
LD	0.458	(0.208–0.977)	0.046
CO_0 day	0.014	(0–0.908)	0.048

CO—carbon oxide, CO_0 day—carbon oxide on the day of stroke onset (before MT), DM—diabetes mellitus, LD—lipid disorders, MT—mechanical thrombectomy, NIHSS—National Institutes of Health Stroke Scale; NIHSS_2—NIHSS on the 2nd day of stroke, TICI—thrombolysis in cerebral infarction.

**Table 3:** Binary regression analysis of the influence of clinical phenodata on the worsened functional status of patients (>2 points on modified Rankin Scale) on the 90th day of stroke.

Parameter	OR	95% CI	*p*
Age	1.065	(1.018–1.123)	0.011
NIHSS_2	1.633	(1.328–2.125)	0.000
sICB	0.232	(0.047–0.992)	0.056

sICB—symptomatic intracranial bleeding, NIHSS—National Institutes of Health Stroke Scale; NIHSS_2—NIHSS on the 2nd day of stroke.

**Table 4:** Binary regression analysis of the influence of clinical phenodata on the death on the 10th day of stroke.

Parameter	OR	95% CI	*p*
NIHSS	1.271	(1.153–1.422)	<0.001
sICB	4.080	(1.752–9.763)	0.001
SO_2_2_	1.212	1.022–1.474	0.03

NIHSS—National Institutes of Health Stroke Scale on the 1st day of stroke, sICB—symptomatic intracranial bleeding, SO_2_2_—SO_2_ concentration on the 2nd day of stroke.

## Data Availability

The data is unavailable due to privacy or ethical restrictions.

## Supplementary Materials

The following supporting information can be downloaded at: https://www.mdpi.com/article/10.3390/jcm13030746/s1, Figure S1. Receiver operating characteristic (ROC) curve of the regression model analysis of the impact phenodata on a worsened functional status of patients (>2 points on modified Rankin Scale) on the 10th day of stroke. Figure S2. Receiver operating characteristic (ROC) curve of the regression model analysis of the impact phenodata on a worsened functional status of patients (>2 points on modified Rankin Scale) on the 90th day of stroke. Table S1. The concentration of air pollutants (SO_2_, NO_2_, CO, O_3_) collected in the period during all study period. Table S2. The concentration of air pollutants (PM2.5, PM10) collected in the period during all study period. Table S3. The correlation between the concentration of air pollutions and the functional outcomes at discharge, the 30th and the 90th day after stroke onset.
