# Supplementary material for: Direct Exposure to Outdoor Air Pollution Worsens the Functional Status of Stroke Patients Treated with Mechanical Thrombectomy
Source: J Clin Med. 2024 Jan 27;13(3):746. doi: 10.3390/jcm13030746 (PMC10856015; doi:10.3390/jcm13030746)
Supplement: Supplementary file 1 [file jcm-13-00746-s001.zip › jcm-2810408-supplementary.pdf]

Figure S1. Receiver operating characteristic (ROC) curve of the regression model analysis of the impact phenodata on a worsened functional status of patients (>2 points on modified Rankin Scale) on the 10th day of stroke.

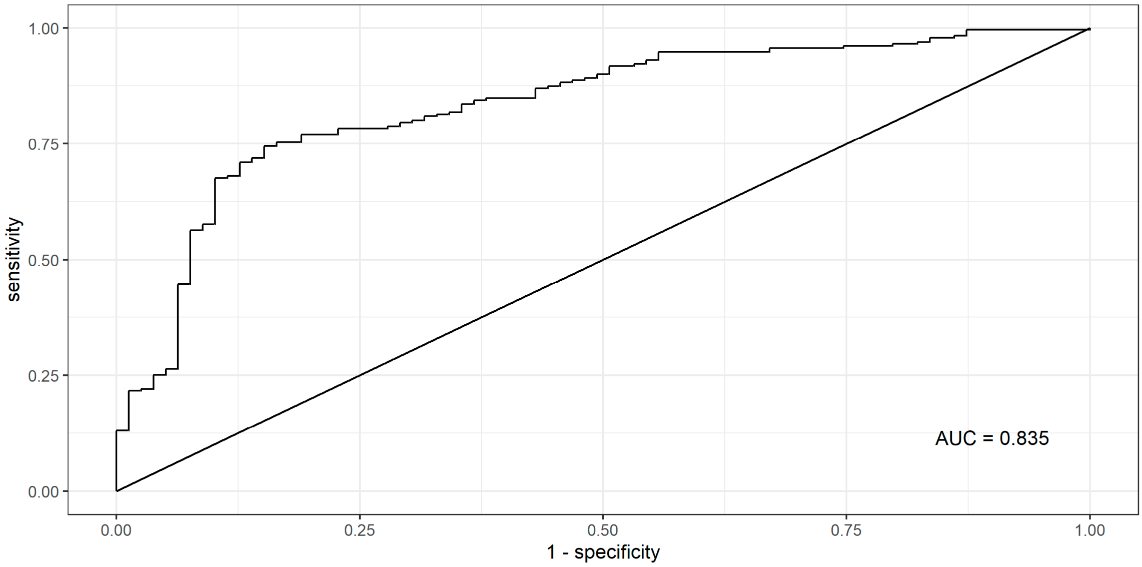

The curve was estimated in a "leave-one-out" procedure and the AUC estimator were used (AUC = 0.835)

Figure S2. Receiver operating characteristic (ROC) curve of the regression model analysis of the impact phenodata on a worsened functional status of patients (>2 points on modified Rankin Scale) on the 90th day of stroke.

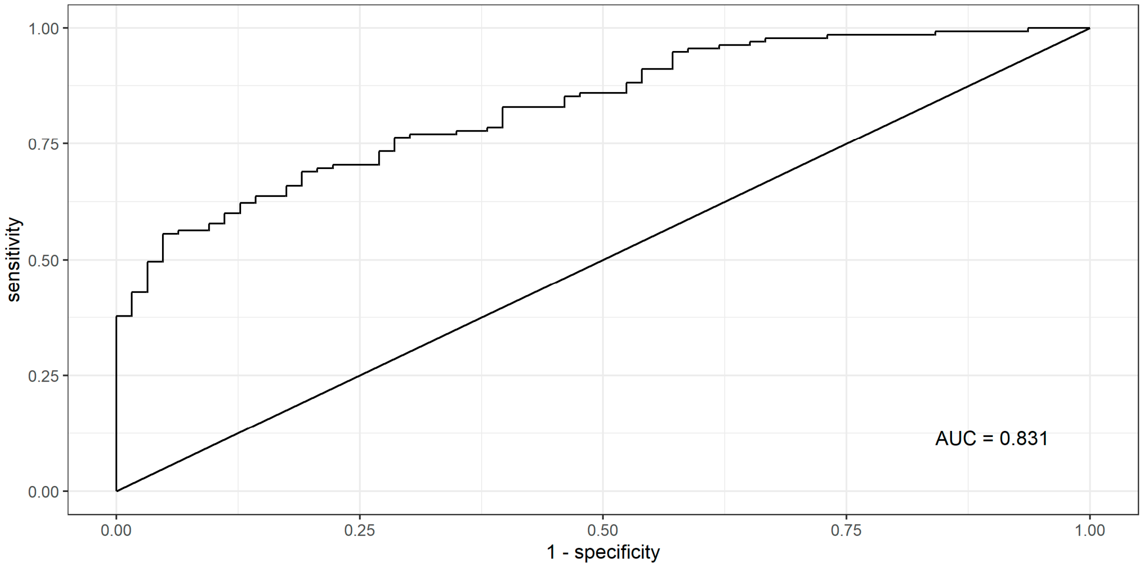

The curve was estimated in a "leave-one-out" procedure and the AUC estimator were used (AUC = 0.831)

Table S1. The concentration of air pollutants (SO2, NO2, CO, O3) collected in the period during all study period.

	2019 (01 JAN- 31 DEC)				2020 (01 JAN-31 DEC)				2021 (01 JAN-31 DEC)				2022 (01 JAN-30 JUN			
	SO2	NO2	CO	O3	SO2	NO2	CO	O3	SO2	NO2	CO	O3	SO2	NO2	CO	O3
DAILY, med±SD, [ref.]	6.58±4.58 [0-23.16]	23.66 ±6.47 [0-55.72]	0.44±0.16 [0.18-1.25]	45.44±19.49 [3.73-90.11]	5.77±5.01 [0-20.11]	23.66 ±6.47 [0-51.02]	0.18±1.17 [0-1.19]	35.24±19.49 [3.73-90.11]	6.78±4.58 [0-23.16]	19.23 ±4.14 [0-53.44]	0.25±0.18 [0-1.42]	31.22±9.31 [2.68-93.47]	5.48±3.58 [0-19.26]	19.23 ±4.14 [0-53.44]	0.09±0.19 [0.72-1.44]	27.47±7.81 [3.72-95.17]
MONTHLY med±SD, [ref.]	6.69±4.48 [0-26.63	24.13 ±7.18 [7.45-53.55]	0.12±0.21 [0-1.16]	45.46±19.81 [2.48-103.11]	6.08±4.22 [0-20.55]	24.13 ±7.18 [4.15-53.55]	0.13±1.19 [0-1.51]	45.46±19.81 [2.48-103.11]	7.69±4.34 [0-25.63]	21.19 ±6.3 [0-54.16]	0.66±0.31 [0-1.43]	40.16±17.12 [1.98-100.01]	6.01±4.12 [0-22.13]	21.19 ±6.3 [0-54.16]	0.14±0.42 [0.61-1.47]	33.13±14.22 [0.88-96.97]
ANNUALLY med±SD, [ref.]	6.98±4.29 [0-26.98]	24.77 ±9.88 [0-56.15	0.1±0.47 [0-1.27]	47.13±18.11 [2.12-107.01]	6.98±5.02 [0-27.43]	25.16 ±8.11 [0-56.15]	0.21±1.48 [0-1.88]	47.13±18.11 [2.12-107.01]	6.98±4.29 [0-27.18]	24.77 ±9.88 [0-58.13	0.11±0.25 [0-1.52]	42.17±16.77 [1.99-103.74]	6.18±3.37 [0-26.98]	24.77 ±9.88 [0-58.13	1.14±0.13 [0.02-1.51]	29.27±14.34 [0.81.99-99.37]

CO - carbon monoxide, NO2 - nitrogen dioxide, O3 - ozone, SO2 - sulfur dioxide

Table S2. The concentration of air pollutants (PM 2.5, PM 10) collected in the period during all study period.

Time range	2019 (01 JAN- 31 DEC)		2020 (01 JAN-31 DEC)		2021 (01 JAN-31 DEC)		2022 (01 JAN-30 JUN	
	PM2.5	PM10	PM2.5	PM10	PM2.5	PM10	PM2.5	PM10
DAILLY, med±SD, [ref.]	20.81±13.68 [0-76.33]	30.04±17.86 [0-98.79]	21.05±15.19 [0-81.24]	28.04±15.82 [0-112.9]	19.89±15.58 [0-87.23]	30.50±19.88 [0-98.13]	22.88±14.66 [0-73.98]	29.74±15.77 [0-87.14]
MONTHLY med±SD, [ref.]	21.28±14.6 [0-78.23]	30.71±18.90 [0-105.25]	20.35±12.83 [0-87.11]	26.08±16.82 [0-119.77]	22.27±12.8 [0-93.23]	24.76±19.34 [0-112.99]	24.24±16.6 [0-80.23]	31.11±17.53 [0-99.24]
ANNUALLY med±SD, [ref.]	20.71±13.69 [0-80.91]	30.38±18.35 [0-123.22]	21.87±13.01 [0-91.93]	317.71±19.44 [0-126.12]	21.28±12.36 [0-103.33]	29.76±17.30 [0-136.92]	24.11±18.14 [0-82.57]	30.76±189.31 [0-105.23]

PM - particulate matter (PM2.5 - particles with aerodynamic diameter <2.5 µm; PM10 - particles with aerodynamic diameter <10 µm)

Table S3. The correlation between the concentration of air pollutions and the functional outcomes at discharge, the 30th and the 90th day after stroke onset.

contaminant	mRS>2 discharge		mRS>2 30th day		mRS>2 90th day	
	Correlation coefficient	p.value	Correlation coefficient	p.value	Correlation coefficient	p.value
PM_2_5_0day	0.91	0.09	0.94	0.06	0.84	0.16
PM_2_5_1day	0.96	0.04	0.94	0.06	0.96	0.04
PM_2_5_3day	0.77	0.23	0.80	0.20	0.85	0.15
PM_2_5_5day	0.73	0.27	1.00	<0.01	0.63	0.37
PM_2_5_8day	0.32	0.68	0.20	0.80	0.18	0.82
PM_10_0day	0.84	0.16	0.88	0.12	0.80	0.20
PM_10_1day	0.84	0.16	0.74	0.26	0.84	0.16
PM_10_3day	0.42	0.58	0.80	0.20	0.95	0.05
PM_10_5day	0.89	0.11	0.67	0.33	0.38	0.62
PM_10_8day	0.71	0.29	1.00	<0.01	0.71	0.29
NO2_0day	0.88	0.12	0.72	0.28	0.80	0.20
NO2_1day	0.89	0.11	0.89	0.11	0.91	0.09
NO2_3day	0.57	0.43	0.42	0.58	0.53	0.47
NO2_5day	0.63	0.37	0.89	0.11	0.55	0.45
NO2_8day	0.69	0.34	0.45	0.55	0.73	0.27
SO2_0day	-0.14	0.86	-0.25	0.75	-0.12	0.88
SO2_1day	0.55	0.45	0.77	0.23	0.89	0.11
SO2_3day	0.00	1.00	0.89	0.11	0.63	0.37
SO2_5day	0.55	0.45	0.30	0.70	0.55	0.45
SO2_8day	0.77	0.23	0.75	0.25	0.63	0.37
CO_0day	0.86	0.14	0.87	0.13	0.84	0.16
CO_1day	0.91	0.09	0.74	0.26	0.63	0.37
CO_3day	0.85	0.15	0.80	0.20	0.55	0.45
CO_5day	0.63	0.37	0.30	0.70	0.18	0.82
CO_8day	-0.91	0.09	-0.75	0.25	-0.73	0.27
O3_0day	-0.60	0.40	-0.25	0.75	0.00	1.00
O3_1day	-0.45	0.55	0.40	0.60	0.60	0.40
O3_3day	0.95	0.05	0.40	0.60	0.18	0.82
O3_5day	0.00	1.00	0.20	0.80	0.32	0.68
O3_8day	0.60	0.40	0.89	0.11	0.84	0.16
